# Lattice-distortion Induced Magnetic Transition from Low-temperature Antiferromagnetism to High-temperature Ferrimagnetism in Double Perovskites *A*_2_FeOsO_6_ (A = Ca, Sr)

**DOI:** 10.1038/srep13159

**Published:** 2015-08-20

**Authors:** Y. S. Hou, H. J. Xiang, X. G. Gong

**Affiliations:** 1 Key Laboratory of Computational Physical Sciences (Ministry of Education), State Key Laboratory of Surface Physics, and Department of Physics, Fudan University, Shanghai 200433, People’s Republic of China; 2 Collaborative Innovation Center of Advanced Microstructures, Nanjing, 210093, People’s Republic of China

## Abstract

High-temperature insulating ferrimagnetism is investigated in order to further reveal its physical mechanisms, as well as identify potentially important scientific and practical applications relative to spintronics. For example, double perovskites such as Sr_2_FeOsO_6_ and Ca_2_FeOsO_6_ are shown to have puzzling magnetic properties. The former is a low-temperature antiferromagnet while the latter is a high-temperature insulating ferrimagnet. In order to understand the underlying mechanisms, we have investigated the frustrated magnetism of *A*_2_FeOsO_6_ by employing density functional theory and maximally-localized Wannier functions. We find lattice distortion enhances the antiferromagnetic nearest-neighboring Fe-O-Os interaction, however weakens the antiferromagnetic interactions via the Os-O-O-Os and Fe-O-Os-O-Fe paths, so is therefore responsible for the magnetic transition from the low-temperature antiferromagnetism to the high-temperature ferrimagnetism as the decrease of the *A*^2+^ ion radii. Also discussed is the 5*d*^3^-3*d*^5^ superexchange. We propose that such superexchange is intrinsically antiferromagnetic instead of ferromagnetic as previously thought. Our work clearly illustrates the magnetic frustration can be effectively relieved by lattice distortion, thus paving the way for tuning of complex magnetism in yet other 3*d*–5*d* (4*d*) double perovskites.

Double perovskite oxides A_2_BB'O_6_, where A is an alkaline-earth or rare-earth metal and B(B') is a transition metal (TM), have attracted considerable attention due to many recent experimental findings, including those related to room-temperature (RT) half-metallicity^1^,^2^,^3^,^4^,^5^, high-temperature (HT) insulating ferrimagnetism^6^,^7^,^8^,^9^,^10^, multiferroicity^11^,^12^,^13^,^14^, ferromagnetism^15^,^16^,^17^ and so on. Recently, the double perovskites Ca_2_FeOsO_6_, SrCaFeOsO_6_ and Sr_2_FeOsO_6_ were found to display dramatically different magnetic behavior^7^,^18^,^19^,^20^,^21^,^22^. Ca_2_FeOsO_6_ is an insulating ferrimagnet with a very high transition temperature of 320 K^7^. If half of the Ca^2+^ ions of Ca_2_FeOsO_6_ were replaced with larger Sr^2+^ ions, the resulting SrCaFeOsO_6_ was found to retain its ferrimagnetic property, but with a significantly lower Curie temperature of 210 K^18^. However, Sr_2_FeOsO_6_ was experimentally found to be an antiferromagnet with low transition temperatures^21^. Experimental observations show that, with the lowering of temperature, Sr_2_FeOsO_6_ transforms from the paramagnetic phase to the AF1 antiferromagnetic phase at 140 K, and then to the AF2 antiferromagnetic phase at 67 K^21^. These experimental results are particularly interesting because these compounds have similar chemical composition. In this context, many important questions remain.

As a result of spin-lattice coupling, the magnetism is usually correlated with the detailed lattice structure. Previous experiments showed that Ca_2_FeOsO_6_, SrCaFeOsO_6_ and Sr_2_FeOsO_6_ have somewhat different lattice distortion patterns. Ca_2_FeOsO_6_ crystallizes with a monoclinic space group of *P2*_1_/n^7^, yet Sr_2_FeOsO_6_ crystallizes with a tetragonal symmetry^20^,^21^. In the *ab* plane, the Fe-O-Os, Os-O-O-Os, Fe-O-Os-O-Fe and Os-O-Fe-O-Os paths of Ca_2_FeOsO_6_ are very similar to those of Sr_2_FeOsO_6,_ except that the lattice distortion in the former case is much stronger (see Fig. 1). In Ca_2_FeOsO_6_, the out-of-plane Fe-O-Os paths are very bent (see Fig. 1a). Consequently, the out-of-plane Fe-O-Os-O-Fe and Os-O-Fe-O-Os paths are highly distorted as well (see Fig. 1a). However, the out-of-plane Fe-O-Os angles in Sr_2_FeOsO_6_ are all nicely 180 degrees, and the out-of-plane Fe-O-Os-O-Fe and Os-O-Fe-O-Os paths are not at all distorted (see Fig. 1b). Compared to Ca_2_FeOsO_6_, Sr_2_FeOsO_6_ also has less distorted out-of-plane Os-O-O-Os paths. Finally, it is worth noting that SrCrFeOsO_6_ takes on a similar structure to Ca_2_FeOsO_6,_ but with a reduced structural distortion^7^,^18^. Therefore, we find progressively weaker lattice distortion when comparing Ca_2_FeOsO_6_ to SrCaFeOsO_6_, to Sr_2_FeOsO_6_.

Although it was pointed out^7^ that lattice distortion is correlated with magnetic behavior, the detailed microscopic mechanism remains unclear. Since the generalized double-exchange mechanism operates only in metals^23^, it cannot account for the HT insulating ferrimagnetism in Ca_2_FeOsO_6_. On the other hand, the origin of AF1 and AF2 spin orders of Sr_2_FeOsO_6_ remains under debate. For the AF1 order, it is widely accepted that the ferrimagnetic (FIM) *ab* planes are coupled to the neighboring planes by the out-of-plane ferromagnetic (FM) Fe-O-Os superexchange^20^,^22^. However, it has been recently suggested that these FIM *ab* planes may be coupled by out-of-plane antiferromagnetic (AFM) Os-O-O-Os interactions^18^. For the AF2 order, Morrow *et al.* proposed that the long-range Fe-Fe AFM interaction (via the four-bond Fe-O-Os-O-Fe path) dominates, and produces AFM-type Fe-Os chains along the *c* axis^18^, however Kanungo *et al.* showed that the long-range Os-Os AFM interaction through the four-bond Os-O-Fe-O-Os path is primarily responsible^22^. For the magnetic ordering temperature, there is to date no clear quantitative understanding as to why the *T*_N_ of AF1 is unexpectedly low. To the best of our knowledge, there also remains a lack of clear understanding as to how LT antiferromagnetism of *A*_2_FeOsO_6_ transforms to HT ferrimagnetism with an accordant increase in lattice distortion, namely, with a decrease in the ionic radii of *A*^2+^ ions.

In this Report, in order to obtain a comprehensive insight into the magnetic behaviors of A_2_FeOsO_6_ (A = Ca, Sr), we systematically investigate the frustrated magnetism of the double perovskites Ca_2_FeOsO_6_, SrCaFeOsO_6_ and Sr_2_FeOsO_6,_ by employing density functional theory (DFT) and maximally-localized Wannier functions (MLWFs). We find lattice distortion enhances the AFM Fe-O-Os interaction but weakens the AFM interactions of the Os-O-O-Os and Fe-O-Os-O-Fe paths. As a result of the serious lattice distortion, Ca_2_FeOsO_6_ has a strong and dominant AFM interaction between the nearest neighboring (NN) Fe^3+^ and Os^5+^ ions. Consequently, the NN Fe^3+^ and Os^5+^ ions are coupled antiparallel and ferrimagnetism is experimentally observed. Simultaneously, corresponding AFM interactions via the Os-O-O-Os and Fe-O-Os-O-Fe paths are weak, so the Os-Os and Fe-Fe induced magnetic frustration is effectively relieved, and one observes a very high *T*_*C*_. Because SrCaFeOsO_6_ is less distorted compared to Ca_2_FeOsO_6_, its magnetic frustration becomes stronger despite the FIM ground state being preserved. Accordingly, its *T*_*C*_ is lowered. In the tetragonal *I*4/m structure of Sr_2_FeOsO_6_, lattice distortion vanishes along the *c* axis but it is very similar to that of Ca_2_FeOsO_6_ in the *ab* plane. This special lattice distortion pattern results in both the in-plane NN Fe^3+^ and Os^5+^ ions being aligned antiparallel and the FM chains along the *c* axis. The resulting magnetic structure is just the strongly frustrated antiferromagnetism AF1 with a very low Neel temperature 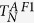. Lastly, strong spin-lattice coupling leads to a transformation from AF1 to AF2. Our work illustrates the magnetic frustration can be effectively relieved by lattice distortion, which may well be responsible for the complex magnetism observed in other 3*d*–5*d* (4*d*) double perovskites as well.

## Results

### Lattice-Distortion dependence of magnetic interactions in Ca_2_FeOsO_6_

**Figure 2:**
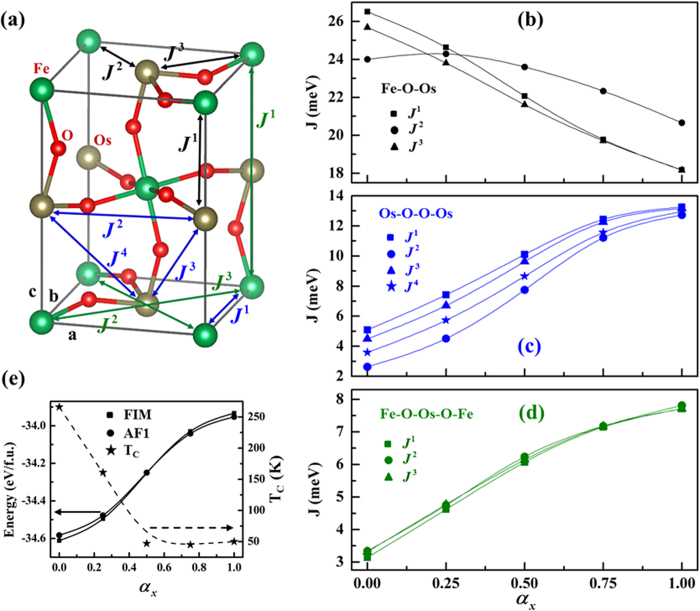
Magnetic exchange paths and evolutions of magnetic interactions and *T*_C_ of Ca_2_FeOsO_6_. (**a**) The NN superexchange paths Fe-O-Os (marked by the black *J*^1^, *J*^2^ and *J*^3^), NNN super-superexchange paths Os-O-O-Os (marked by the blue *J*^1^, *J*^2^, *J*^3^ and *J*^4^) and long-range four-bond Fe-O-Os-O-Fe exchange paths 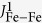, 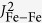 and 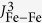 (marked by the green *J*^1^, *J*^2^, *J*^3^ and *J*^4^) are shown by the black, blue and green solid lines, with double arrowheads respectively. The dependence of Fe-O-Os superexchange interactions, Os-O-O-Os super-superexchange interactions, and the long-range four-bond Fe-O-Os-O-Fe magnetic interactions on the lattice distortion (*α*_*x*_) are shown in (**b**–**d**), respectively. Figure (**e**) shows the evolution of *T*_C_ (star) obtained by Monte Carlo and the energy of the FIM (square) and AF1 (circle) magnetic structures with respect to lattice distortion (*α*_*x*_).

In order to understand why Ca_2_FeOsO_6_ is FIM, and how lattice distortion affects this ferrimagnetism, we have systematically explored the effect of lattice distortion on the magnetic interaction of Ca_2_FeOsO_6_. Since the positions of O^2−^ ions are known to be vital, we performed a series of calculations using a linear superposition of the Wyckoff positions of the O^2−^ ions of both the relaxed and pseudo-cubic structure. In the pseudo-cubic structure, O^2−^ ions are artificially positioned to make Fe-O-Os angles straight, but lattice constants and the positions of the Fe^3+^, Os^5+^ and Ca^2+^ ions are fixed at their corresponding positions in the relaxed structure. The O^2−^ ions positions is computed as follows:
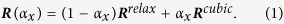


In Eq. (1), ***R***^*relax*^ and ***R***^*cubic*^ are the position vectors of O^2−^ ions in the relaxed and pseudo-cubic structures respectively, and *α*_*x*_ varies between 0 and 1. For example, *α*_*x*_ = 0 corresponds to the relaxed structure and *α*_*x*_ = 1 corresponds to the pseudo-cubic structure. Thus *α*_*x*_ characterizes the lattice distortion induced by O^2−^ ions. The dominant magnetic interactions are divided into three groups (see Fig. 2a). The first group is the superexchange between the NN Fe^3+^ and Os^5+^ ions. The second involves super-superexchange between the next near-neighboring (NNN) Os^5+^ ions. The third involves long-range Fe-Fe interactions via the four-bond Fe-O-Os-O-Fe path. Technically, we adopt the four-state mapping method to evaluate these various magnetic interactions^24^. Note that a positive exchange constant *J* corresponds to the AFM interaction, but a negative exchange constant *J* corresponds to the FM interaction.

We find the magnetic interaction between the NN Fe^3+^ and Os^5+^ ions is intrinsically AFM. The calculated magnetic exchange constants of Fe-O-Os paths in the pseudo-cubic structure are shown in the Fig. 2b. They are all positive and thus AFM. The intrinsically AFM interaction of the Fe-O-Os path can be qualitatively understood based upon the extended Kugel-Khomskii model^25^,^26^,^27^. According to this model, magnetic interactions can be evaluated based on the hopping integrals and on-site energies, namely,
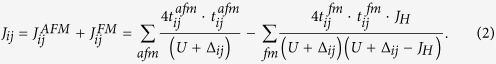


In Eq. (2), *U*, *J*_*H*_ and Δ_*ij*_ are the on-site Coulomb interaction, Hund’s coupling and the energy difference between the i^th^ and j^th^ energy levels, respectively, and 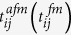 is the hopping integral. The first term in *J*_*ij*_ describes the AFM contribution due to the hybridization between the two occupied orbitals. The second term describes the FM contribution due to the hybridization between the occupied and empty orbitals. In order to elucidate why the magnetic interaction between the NN Fe^3+^ and Os^5+^ ions is intrinsically AFM, we take the Fe-O-Os path along the *c* axis of the pseudo-cubic Ca_2_FeOsO_6_ as a typical example. Its detailed hopping integrals and energy levels are given in the right panel of Fig. S1 of supplemental material (SM). Compared with the FM interaction between the NN Mn^3+^ ions in the cubic LaMnO_3_ (LMO)^28^, two pivotal factors are seen to drive the magnetic interaction between the NN Fe^3+^ and Os^5+^ ions in the pseudo-cubic Ca_2_FeOsO_6_ to be intrinsically AFM. The first factor is the very large energy difference Δ (up to 3.0 eV) between the occupied *e*_*g*_ orbitals of Fe^3+^ ion and the unoccupied *e*_*g*_ orbitals of Os^5+^ ion. This will give a weak FM contribution according to the Eq. (2). The second factor is the rather large hopping integrals between the occupied *t*_2*g*_ orbitals of the Fe^3+^ and the Os^5+^ ions. For instance, the leading hopping integral is 0.27 eV. This will give strong AFM contribution according to the Eq. (2). Therefore the AFM contribution dominates over the FM one, giving rise to the intrinsically AFM interaction between the NN Fe^3+^ and Os^5+^ ions, regardless of the magnitude of the Fe-O-Os angle.

In addition, we find lattice distortion can effectively relieve the magnetic frustration in Ca_2_FeOsO_6_ and thereby raise its FIM phase transition temperature *T*_*C*_. Since Os^5+^ ions form a face-centered sublattice with geometrically frustrated edge-sharing tetrahedrons, antiferromagnetically interacting Os^5+^ ions are strongly frustrated. Figure 2c shows lattice distortion can dramatically weaken the NNN AFM interactions between the NNN Os^5+^ ions, which implies that the Os^5+^ ions’ induced magnetic frustration can be relieved by lattice distortion. Besides, Fe^3+^ ions can also be magnetically frustrated because of the following factor. The dominant NN Fe-O-Os AFM interactions require the magnetic moments of Fe^3+^ ions to be aligned parallel, but the AFM magnetic interaction through the four-bond Fe-O-Os-O-Fe paths requires the magnetic moments of Fe^3+^ ions to be antiparallel. Because lattice distortion slightly enhance the NN AFM interactions between the NN Fe^3+^ and Os^5+^ ions (see Fig. 2b) but weakens the long-range four-bond Fe-O-Os-O-Fe AFM interactions along the pseudo-cubic [001], [010] and [100] axes (see Fig. 2d), it can effectively relieve the Fe^3+^ ions induced magnetic frustration. And, we should note, accompanied with the relief of such magnetic frustration is the raising of the FIM phase transition temperature *T*_*C*_. Figure 2e shows the evolution of *T*_*C*_ obtained by Monte Carlo (MC) as lattice distortion weakens. It clearly shows the *T*_*C*_ of the relaxed structure 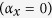 (about 266 K, close to the experimentally measured one^7^ *T*_*C*_ ≈ 320 K) is higher than that of the less distorted one 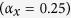. Note that *T*_*C*_ slightly increases with the weakening of lattice distortion for large *α*_*x*_. This is because the magnetic ground state of Ca_2_FeOsO_6_ with small lattice distortion is no longer FIM but AFM with the AF1 order as appearing in the Sr_2_FeOsO_6_ (see Fig. 2e).

**Figure 3:**
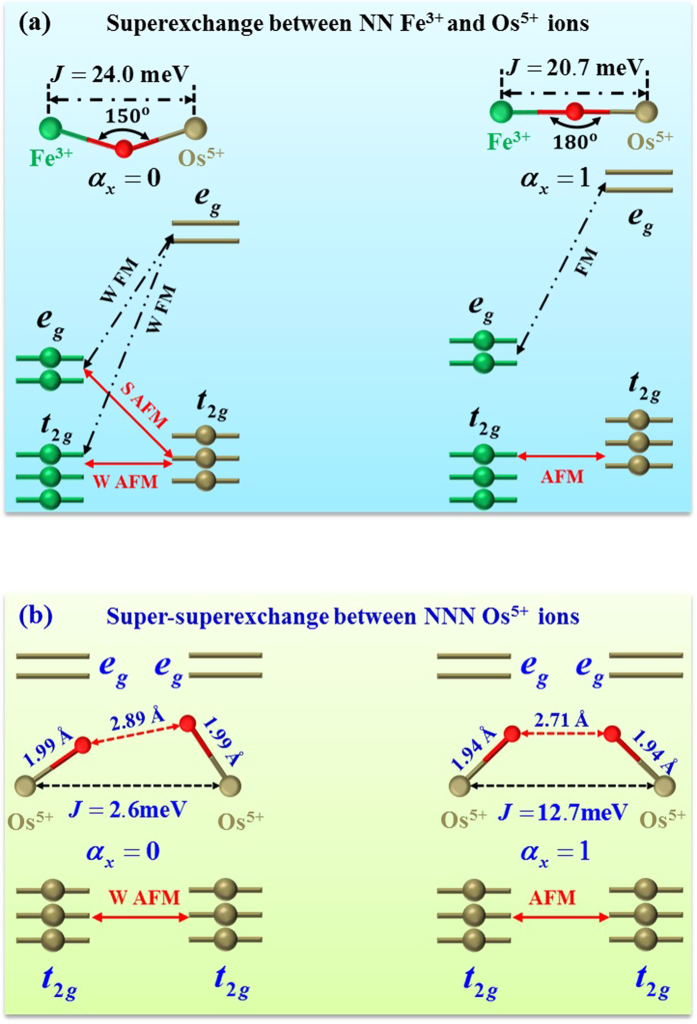
Mechanism by which lattice distortion enhances the NN AFM interaction between Fe^3+^ and Os^5+^ ions (**a**), and weakens the NNN AFM interaction between Os^5+^ ions (**b**) in Ca_2_FeOsO_6_. Solid (dashed) lines with double arrowheads indicate the electron hopping causing AFM (FM) contribution to the NN superexchange or NNN super-superexchange. S and W represent “strong” and “weak” words, respectively. In (**a**), the FM contribution of *a*_*x*_ = 0 is weaker than that of *a*_*x*_ = 1. However, the AFM contribution of *a*_*x*_ = 0 is stronger than that of *a*_*x*_ = 1. In (**b**), the AFM contribution of *a*_*x*_ = 0 is weaker than that of *a*_*x*_ = 1. Insets in (**a**,**b**) are for the local structures of Fe-O-Os and Os-O-O-Os paths, respectively. The relevant bond angles, bond lengths and calculated magnetic exchange constants are explicitly given in the inset of figures (**a**) and (**b**).

Figure 3a demonstrates the mechanism by which lattice distortion enhances the NN Fe-O-Os AFM interaction. For illustration purposes, we consider the Fe-O-Os path along the *c* axis as an example. Fig. S1 of the SM shows the detailed leading hopping integrals and energy levels in the relaxed and pseudo-cubic structures, respectively. These hopping integrals clearly indicate lattice distortion tremendously reduces the electron hopping between the occupied *e*_*g*_ orbitals of Fe^3+^ ions and the unoccupied one of Os^5+^ ions. Consequently, one can conclude based on the formula of *J*_*ij*_ (see Eq. (2)) that lattice distortion extraordinarily reduces the FM contribution to the NN superexchange. In contrast, lattice distortion has a rather minor effect on the AFM contribution, because it increases the electrons hopping between the occupied *e*_*g*_ orbitals of Fe^3+^ ions and the occupied *t*_2*g*_ orbitals of Os^5+^ ions, although it reduces the hopping between the occupied *t*_2*g*_ orbitals of Fe^3+^ and Os^5+^ ions. Therefore, lattice distortion enhances the NN AFM interaction by dramatically reducing the FM contribution, and by maintaining the AFM contribution almost unchanged.

We find the NNN AFM interaction between the NNN Os^5+^ ions is weakened by lattice distortion. This is because such NNN super-superexchange has a sensitive dependence on the geometry of the Os-O-O-Os path. Shown in the insets of Fig. 3b are the geometries of the relevant Os-O-O-Os paths in the relaxed and pseudo-cubic structures. The detailed leading hopping integrals and energy levels between the investigated Os^5+^ ions are shown in the Fig. S2 of the SM. Note that FM contribution to the NNN super-superexchange is rather weak in the relaxed and pseudo-cubic structures because of small hopping integrals and large energy differences. So the NNN super-superexchange is basically determined by the AFM contribution. Comparison of the two investigated Os-O-O-Os paths shown in Fig. 3b clearly indicates lattice distortion increases the O-O bond length. Such an increase can reduce the hopping between the *t*_2*g*_ electrons of Os^3+^ ions, as is readily verified by the reduction of hopping integrals from the pseudo-cubic structure to the relaxed one (see Fig. S2 of the SM). Therefore lattice distortion blocks the *t*_2*g*_ electron hopping through Os-O-O-Os path, thereby weakening the otherwise robust NNN AFM interaction.

### Low-temperature antiferromagnetism of Sr_2_FeOsO_6_

**Figure 4:**
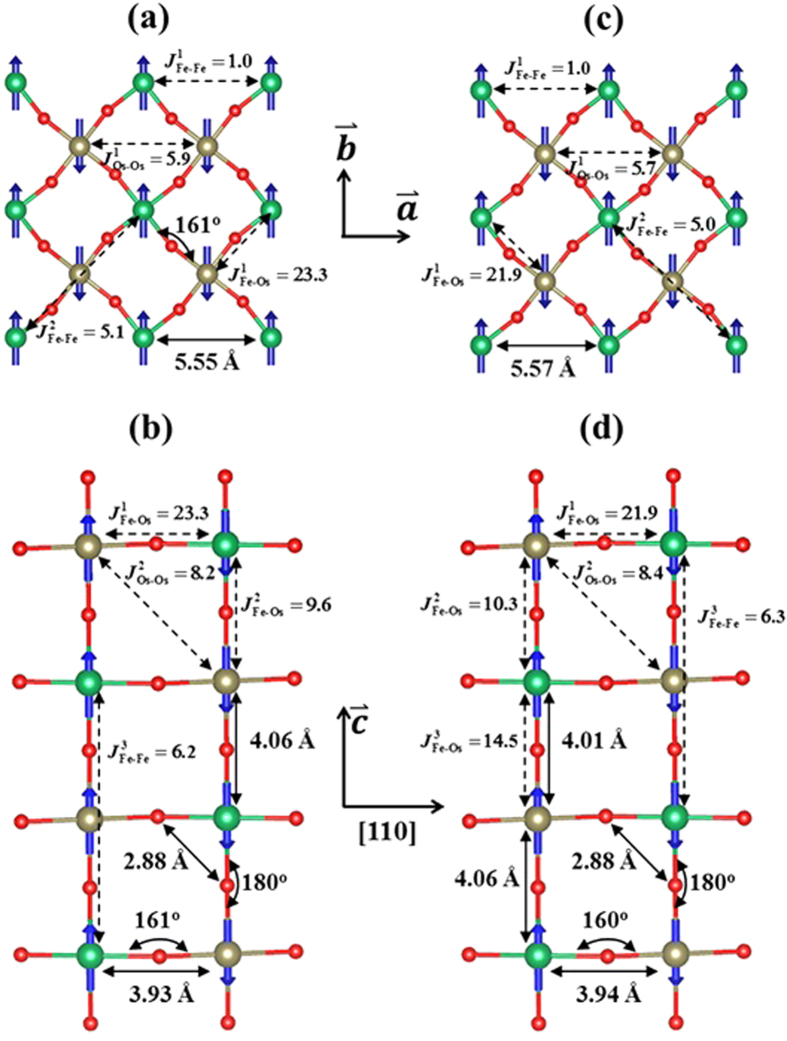
Dominant magnetic exchange paths and AF1, AF2 magnetic structures of Sr_2_FeOsO_6_. All magnetic exchange constants *J* are in units of meV. Figures (**a**,**b**) correspond to the *I*4/m-AF1 phase. Figures (**c**,**d**) correspond to the *I*4-AF2 phase. Figures (**a**,**c**) are the spin arrangement in the tetragonal *ab* plane. Figures (**b**,**d**) are the spin ordering along the *c* axis. Blue arrows represent spins. The relevant bond distances and angles obtained from DFT calculations are shown in (**a**–**d**).

Sr_2_FeOsO_6_ adopts two different magnetic and lattice structures depending on temperature^21^. With decreasing temperature, its magnetic structure transforms from AF1 into AF2 antiferromagnetism and its lattice structure transforms from *I*4/m into *I*4 with a dimerization between the NN Fe^3+^ and Os^5+^ ions along the *c* axis. In both AF1 and AF2, moments of Fe^3+^ and Os^5+^ ions are coupled antiparallel in the *ab* plane (Fig. 4a and Fig. 4c). In AF1 spins order as ++++ along the *c* axis (Fig. 4b). In AF2, spins order as ++−−++−− (Fig. 4d).

Our study on the *I*4/m-AF1 phase (Fig. 4a and Fig. 4b) shows that the out-of-plane NN AFM interaction 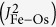 is much weaker than its in-plane counterpart 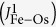 and that the out-of-plane NNN AFM interaction 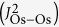 is stronger than the in-plane counterpart 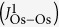, which are readily understood based upon our above results for Ca_2_FeOsO_6_. Because the Fe-O-Os angle along the *c* axis is 180.0°, similar to that in pseudo-cubic Ca_2_FeOsO_6_, the out-of-plane NN AFM interaction 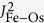 is weak. In the tetragonal *ab* plane, lattice distortion is similar to that in the relaxed Ca_2_FeOsO_6_, so the in-plane NN AFM interaction 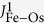 is strong. The weak in-plane NNN AFM interaction 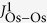 is due to the strong in-plane lattice distortion blocking the Os^5+^ ions’ *t*_2*g*_ electron hopping, similar to the weak NNN AFM interactions in the relaxed Ca_2_FeOsO_6_. Besides, the out-of-plane NNN AFM interaction 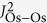 is stronger than that of the relaxed Ca_2_FeOsO_6_ but weaker than that of the pseudo-cubic Ca_2_FeOsO_6_. Overall, such magnitudes of the spin interactions are a result of the combination of the absence of the lattice distortion along the *c* axis and the strong lattice distortion in the tetragonal *ab* plane. Finally, it is expected that the long-range four-bond Fe-O-Os-O-Fe AFM interaction along the *c* axis 
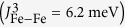
 is stronger than the in-plane counterpart 
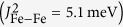
. Note that the super-superexchange Fe-Fe interaction 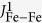 through the Fe-O-O-Fe path is very weak 
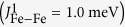
 compared with the others, and thus is omitted from our model.

**Figure 5:**
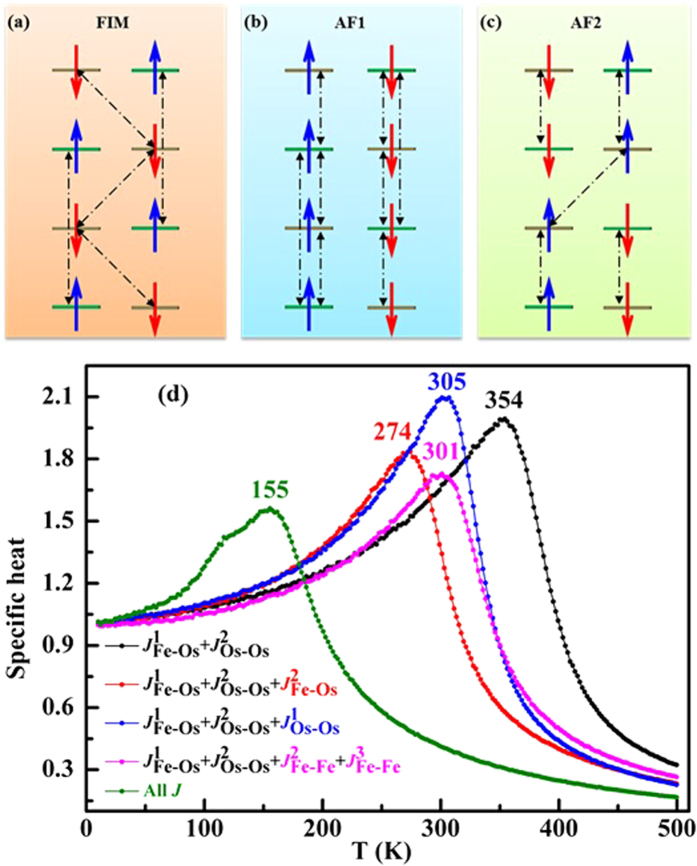
FIM (**a**), AF1 (**b**) and AF2 (**c**) magnetic structures. In (**a**–**c**), the frustrated magnetic ions pairs are connected by black dashed lines with double arrowheads. Fe (Os) sites are represented by the green (gray) horizontal lines. Blue (red) arrows represent up (down) spins. Figure (**d**) shows the specific heat of Sr_2_FeOsO_6_, calculated as a function of temperature T in terms of spin exchange interactions. The peak locates the magnetic phase transition temperature *T*_*N*_.

Here we discuss how the competing magnetic interactions establish AF1 in the tetragonal *I*4/m structure of Sr_2_FeOsO_6_. First, it should be noted that the magnetic easy axis is the *c* axis^21^, that is, magnetic moments can only point up and down along it. The calculated results (see Fig. 4a) show that the in-plane NN AFM interaction 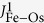 is approximately four times of the magnitude of the in-plane NNN AFM interaction 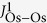, as well as the in-plane long-range four-bond Fe-O-Os-O-Fe AFM interaction 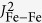. In addition, their pairwise numbers (Z’s) are all the same (*Z* = 4). For the in-plane magnetic interactions, therefore, the optimal configuration is such that the magnetic moments of Fe^3+^ and Os^5+^ ions are aligned antiparallel in the *ab* plane, as experimental observations^21^. For the magnetic interaction along the *c*-axis, the out-of-plane NN 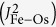, NNN 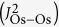 and the long-range four-bond 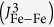 magnetic interactions are all AFM (see Fig. 4b). If only the out-of-plane NN AFM interaction 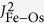 is taken into consideration, the FIM Fe^3+^-Os^5+^ layers should be coupled antiparallel along the *c* axis. In this case, the resulting magnetic structure is FIM (see Fig. 5a). If only the out-of-plane NNN AFM interaction 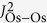 is taken into consideration, the FIM Fe^3+^-Os^5+^ layers should be coupled parallel along the *c* axis. In this case, the resulting magnetic structure is AF1 (Fig. 5b), which is just the experimentally observed. Finally, if only the long-range four-bond Fe-O-Os-O-Fe AFM interaction 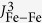 is taken into consideration, it gives rise to AF2 (Fig. 5c). Obviously, the out-of-plane NN 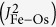, NNN 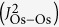 and the long-range four-bond 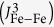 AFM interactions compete to give rise to the different magnetic ground states. Since their magnitudes are comparable, their pairwise numbers are the decisive factor in determining the optimal magnetic structure, being *Z*_*NN*_ = 2, *Z*_*NNN*_ = 8 and 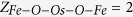, respectively. This indicates the out-of-plane NNN AFM interaction 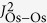 easily dominates the out-of-plane NN 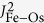 and long-range four-bond 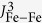 AFM interactions. Therefore the optimal magnetic configuration is AF1.

This deduction can be confirmed as follows. In the FIM, all the out-of-plane Fe-Fe and Os-Os pairs are frustrated (see Fig. 5a). In the AF1, all the out-of-plane Fe-Os and Fe-Fe pairs are frustrated (see Fig. 5b). In the AF2, half of the out-of-plane Fe-Os and Os-Os pairs are frustrated (see Fig. 5c). In terms of the out-of-plane NN 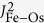, NNN 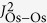 and long-range four-bond 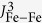 AFM interactions, therefore, the formula-unit (f.u.) magnetic energies of the FIM, AF1 and AF2 are as follows:
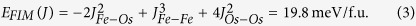

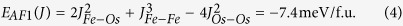

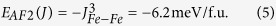


This indicates that FIM should have the highest energy, with AF2 at a median value, then AF1 at the lowest level. Such estimation is in accord with our DFT calculations: 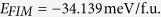 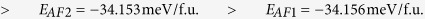 So AF1 is found to readily relieve magnetic frustration.

The low Neel temperature *T*_N_ of the AF1 is a result of the strong magnetic frustration. Actually, only the in-plane 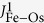 and the out-of-plane 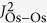 AFM interactions are not frustrated in the AF1. However, the in-plane 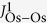, out-of-plane 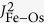 and the long-range four-bond 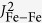, 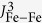 AFM interactions antagonize the AF1 antiferromagnetism, and therefore will induce frustration. Our MC simulations (see Fig. 5d) indicate the *T*_N_ of AF1 is very high, up to 354 K, sharply contradicting with the experimentally observed value (140 K), if only 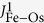 and 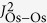 are taken into consideration. To determine why the experimentally measured *T*_N_ is so low, we performed four additional MC simulations: one with the in-plane 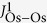, one with the out-of-plane 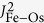, one with the long-range four-bond 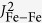 and 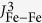, and one with all of these magnetic interactions. The resulting specific heat versus temperature plots are presented in Fig. 5d. As can be seen, 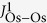, 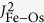 and the long-range four-bond 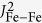 and 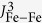 can all lower the *T*_N_ because they are all frustrated. Moreover, the out-of-plane Fe-O-Os AFM interactions make the largest contribution to the lowering of *T*_N_ for AF1. If all the dominating magnetic interactions are taken into consideration, the MC simulated *T*_N_ is 155 K, which is very close to the experimental value.

By comparing the magnetic exchange constants of *I*4 structure with those of *I*4/m structure (see Fig. 4), one finds that the magnetic interactions in the former are very similar to the latter’s, with the exception that the rather slight dimerization along the *c* axis in the *I*4 structure prominently enhances the out-of-plane NN AFM interactions 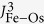 (see Fig. 4d), which indicates a very strong spin-lattice coupling. Like in the *I*4/m structure, the long-range four-bond Fe-O-Os-O-Fe AFM interaction 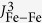 favors the formation of the AF2, as does the enhancement of the out-of-plane 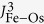. Thus we attribute the AF2 antiferromagnetism in *I*4 structure to the strong spin-lattice coupling.

### Low-temperature ferrimagnetism in the SrCaFeOsO_6_

**Figure 6:**
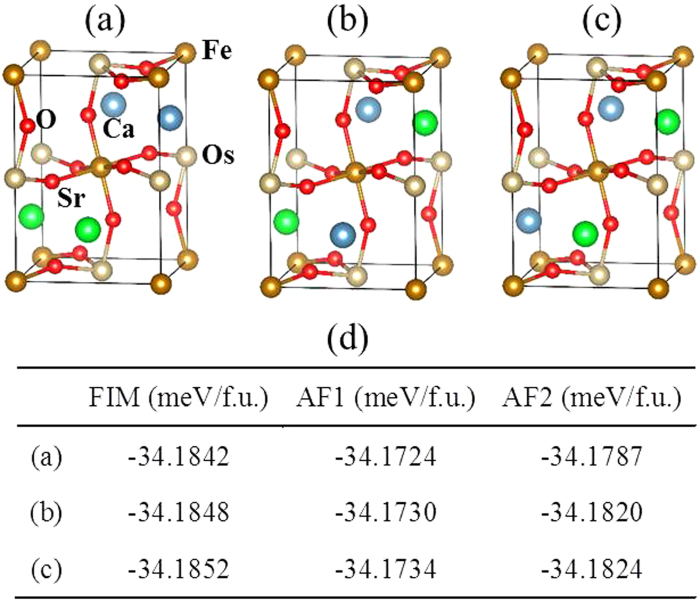
Three types of arrangement patterns of Ca^2+^ and Sr^2+^ ions in the SrCaFeOsO_6,_ and their corresponding energies. (**a**) All Ca^2+^ (Sr^2+^) are arranged in the *ab* plane. (**b**) All Ca^2+^ (Sr^2+^) are arranged along the *c* axis. Figure (**c**) shows Ca^2+^ and Sr^2+^ ions are arranged in the checkerboard manner. Figure (**d**) shows the energies of the FIM, AF1 and AF2 magnetic structures of the three most typical arrangement patterns of the Ca^2+^ and Sr^2+^ ions.

Comparing SrCaFeOsO_6_ with Sr_2_FeOsO_6_ and Ca_2_FeOsO_6_, one can conclude that its mediate lattice distortion causes its ferrimagnetism to have a lower *T*_C_. Experiments show that SrCaFeOsO_6_ has a rather similar lattice structure to that of Ca_2_FeOsO_6_^18^. However, its Fe-O-Os bond angles reveal a more linear geometry than that of Ca_2_FeOsO_6_, because half of Ca^2+^ ions are replaced by larger Sr^2+^ ions^18^. So it can be inferred that SrCaFeOsO_6_ can be readily ferrimagnetic. To confirm this, we studied three different types of arrangements of Ca^2+^ and Sr^2+^ ions. The first is where all the Ca^2+^ (Sr^2+^) are arranged in the *ab* plane (Fig. 6a). The second is where all Ca^2+^ (Sr^2+^) are arranged along the *c* axis (Fig. 6b). The third is where Ca^2+^ and Sr^2+^ ions are arranged in a checkerboard manner (Fig. 6c). For each arrangement, the FIM, AF1 and AF2 are considered. In all three of these cases, FIM always has the lowest total energy (see Fig. 6d). So the magnetic ground of SrCaFeOsO_6_ should be FIM, consistent with experimental observations^18^. Since its Fe-O-Os bond becomes straighter, its NN Fe-O-Os AFM interactions become weaker however its NNN Os-O-O-Os and long-range four-bond Fe-O-Os-O-Fe AFM interactions become stronger. This is verified by the calculated magnetic exchange parameters, as listed in the Table *I* of the SM. Consequently, its magnetic frustration gets stronger and its *T*_C_ should accordingly be lowered. Our MC simulated *T*_C_ for SrCaFeOsO_6_ is approximately 100 K, lower than the corresponding *T*_C_ of 266 K for Ca_2_FeOsO_6_, consistent with experimental observations.

## Discussions

Based on the present work, an important and general rule on the 3*d*^5^–5*d*^3^ superexchange in the double perovskites can be proposed as follows. It is generally accepted that^29^ the *d*^5^-*d*^3^ superexchange changes from FM for *θ* > *θ*_*c*_ to AFM for *θ* < *θ*_*c*_ with 135° < *θ*_*c*_ < 150°. However, we demonstrate the magnetic interaction between 3*d*^5^ and 5*d*^3^ TMs will be intrinsically AFM (this conclusion is independent of the particular choice of (a reasonable) U, see Table *II* of the SM) and further that this AFM interaction will increase as its angle *θ* decreases, as evidenced by the Fe-O-Os interactions in Ca_2_FeOsO_6_, SrCaFeOsO_6_ and Sr_2_FeOsO_6_. This intrinsically AFM interaction results from both the large hopping integrals between the occupied *t*_2*g*_ orbitals and the large energy difference between the occupied *e*_*g*_ orbitals of 3*d* TM, and the unoccupied orbitals of 5*d* TM, because the former gives rise to a strong AFM contribution and the latter gives rise to a relatively weak FM contribution to the 3*d*^5^–5*d*^3^ superexchange. As the angle *θ* decreases, the electron hoppings between the occupied *e*_*g*_ orbitals of the 3*d*^5^ TM and the unoccupied ones of the 5*d*^3^ TM will be substantially reduced, but the electron hopping between the occupied orbitals of 3*d*^5^ TM and 5*d*^3^ TM remain largely unchanged. Thus decreasing the angle *θ* means reducing the FM contribution, while leaving the AFM contribution largely unchanged. Consequently, the AFM interaction of the 3*d*^5^-O-5*d*^3^ path increases with decreasing *θ*.

## Conclusions

In conclusion, we have investigated the effect of lattice distortion on the frustrated magnetism of certain double perovskites: Ca_2_FeOsO_6_, Sr_2_FeOsO_6_ and Sr_2_CrOsO_6_. In these cases, we find lattice distortion enhances the NN AFM Fe-O-Os interactions but weakens the AFM interactions of the Os-O-O-Os and Fe-O-Os-O-Fe paths. Because lattice distortions become increasingly severe from Sr_2_FeOsO_6_ to SrCaFeOsO_6_ to Ca_2_FeOsO_6_, the NN AFM Fe-O-Os interactions also become increasingly strong, but the AFM interactions of Os-O-O-Os and Fe-O-Os-O-Fe paths become increasingly weak. Consequently, the magnetic ground state transforms from antiferromagnetism to ferrimagnetism, and the magnetic transition temperature increases. We propose the 5*d*^3^-3*d*^5^ superexchange is intrinsically antiferromagnetic, instead of being, as previously thought, ferromagnetic. Our work illustrates the magnetic frustration can be effectively relieved by lattice distortion in certain 3*d*–5*d* (4*d*) double perovskites.

## Methods

### First-principles calculations

First-principles calculations based on DFT are performed within the generalized gradient approximation (GGA) according to the Perdew-Burke-Ernzerhof (PBE) parameterization as implemented in Vienna *Ab initio* Simulation Package (VASP)^30^. The projector-augmented wave method^31^, with an energy cutoff of 500 eV and a gamma-centered k-point mesh grid are used. Ion positions are relaxed towards equilibrium with the Hellmann-Feynman forces on each ion set to be less than 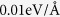. We use the simplified (rotationally invariant) coulomb-corrected density functional (DFT + U) method according to Dudarev *et al.*^32^. 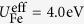, and 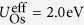 are applied to the Fe 3*d* and Os 5*d* states^22^, respectively. With this effective U, the calculated band gap of Ca_2_FeOsO_6_ is about 1.19 eV, very close to the experimentally measured activation energy^7^ 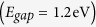. Because the spin-orbit coupling (SOC) in Ca_2_FeOsO_6_ has been demonstrated to be insignificant^19^, SOC is not taken into account in this present work.

### Maximal localized Wannier functions calculations

Hopping integrals between 3*d*/5*d* orbitals are extracted from the real-space Hamiltonian matrix elements in the non-spin-polarized MLWFs basis. MLWFs are obtained by employing the **vasp2wannier90** interface in combination with the **wannier90** tool^33^. In order to obtain the 3*d*/5*d*-like Wannier functions, we construct MLWFs in a suitable energy window, using primarily 3*d*/5*d* antibonding states. All MLWFs are considered to be well converged if the total spread over 50 successive iterations is smaller than 10^−9^ Å^2^.

### Monte Carlo simulations

The magnetic phase transition temperature *T*_*C*_ or *T*_*N*_ is obtained using parallel tempering Monte Carlo simulations^34^,^35^. These calcuations are performed on the 7 × 7 × 5 supercell based on the classical spin Hamiltonian: 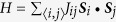, wherein the spin exchange parameters *J*_*ij*_ are those defined in the text. To obtain the *T*_*C*_ or *T*_*N*_, we first calculate the specific heat 
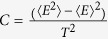
 once the system reaches equilibrium at a given temperature (*T*). Then *T*_*N*_ can be obtained by locating the maximum on the C(*T*) vs *T* plot.

## Additional Information

**How to cite this article**: Hou, Y. S. *et al.* Lattice-distortion Induced Magnetic Transition from Low-temperature Antiferromagnetism to High-temperature Ferrimagnetism in Double Perovskites *A*_2_FeO_s_O_6_ (A=Ca, Sr). *Sci. Rep.* **5**, 13159; doi: 10.1038/srep13159 (2015).

## Supplementary Material

Supplementary Information

## Figures and Tables

**Figure 1:**
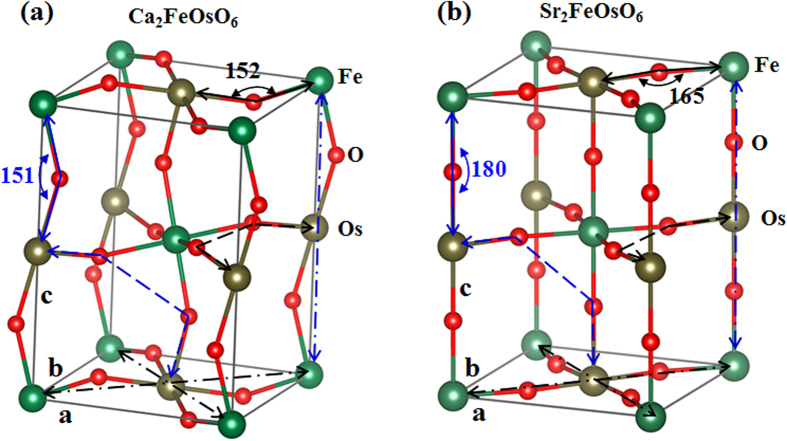
Lattice structures of Ca_2_FeOsO_6_ (**a**) and Sr_2_FeOsO_6_ (**b**). The Fe-O-Os paths are shown by solid lines, and experimentally measured Fe-O-Os angles are also shown in units of degrees. The Os-O-O-Os paths are depicted by dashed lines. The Fe-O-Os-O-Fe and Os-O-Fe-O-Os paths are depicted by dot-dashed lines. The in-plane and out-of-plane paths are shown in black and blue, respectively. The letter **a**, **b** and **c** denote the crystal axes. Ca^2+^ and Sr^2+^ ions are not displayed for the sake of clarity.
