# Supplementary material for: Lattice-distortion Induced Magnetic Transition from Low-temperature Antiferromagnetism to High-temperature Ferrimagnetism in Double Perovskites A2FeOsO6 (A = Ca, Sr)
Source: Sci Rep. 2015 Aug 20;5:13159. doi: 10.1038/srep13159 (PMC4542468; doi:10.1038/srep13159)
Supplement: Supplementary Information [file srep13159-s1.docx]

**Supplemental Material of “Lattice-distortion Induced Magnetic Transition from Low-temperature Antiferromagnetism to High-temperature Ferrimagnetism in Double Perovskites *A*_2_FeOsO_6_ (A=Ca, Sr)”**

Y. S. Hou, H. J. Xiang, and X. G. Gong

Key Laboratory of Computational Physical Sciences (Ministry of Education), State Key Laboratory of Surface Physics, and Department of Physics, Fudan University, Shanghai 200433, People’s Republic of China

1. **Superexchange between the nearest-neighboring (NN) Fe^3+^ and Os^5+^ ions along the *c* axis in Ca_2_FeOsO_6_**


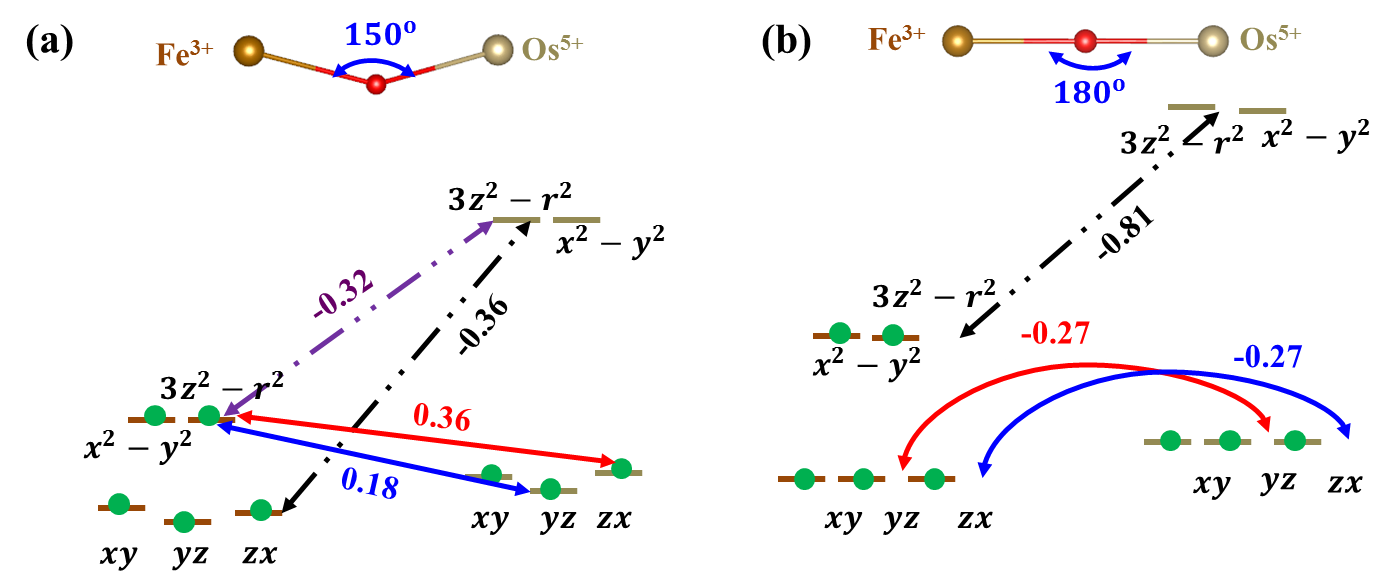


FIG. S1. (Color online) Shown are the energy levels, leading hopping integrals and geometrical structures of the NN superexchange Fe^3+^-Os^5+^ paths. (a) corresponds to the relaxed structure and (b) to the pseudo-cubic structure. All hopping integrals are in units of eV. Green circles represent electrons. Solid (dashed) curves with double arrowheads indicate the electron hopping that makes AFM (FM) contributions to the NN superexchange. Inserts in (a) and (b) are the local geometrical structures of the Fe-O-Os paths.

1. **Super-superexchange between the next nearest-neighboring (NNN) Os^5+^ ions in Ca_2_FeOsO_6_**


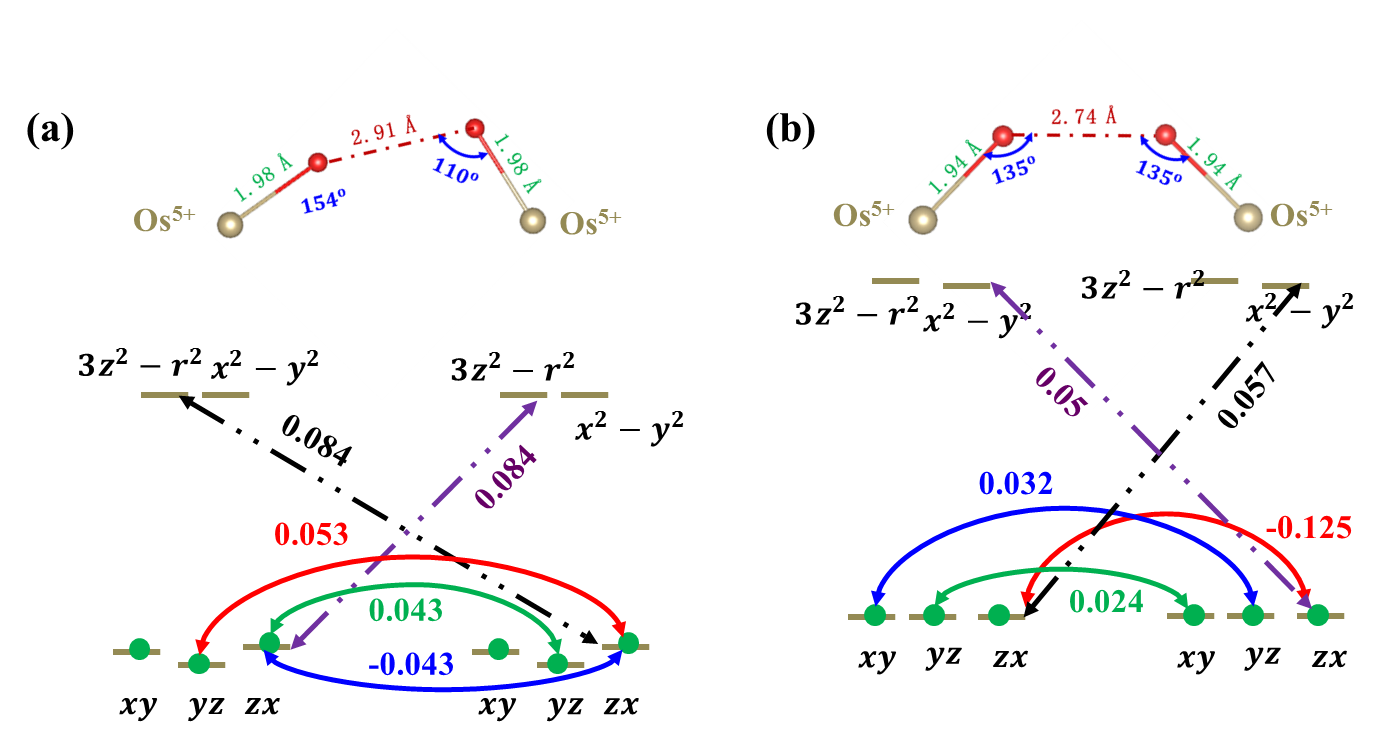


FIG. S2. (Color online) Shown are the energy levels, leading hopping integrals, and geometrical structures of the NNN super-superexchange Os^5+^-Os^5+^ paths. (a) corresponds to the relaxed structure and (b) to the pseudo-cubic one. All hopping integrals are in units of eV. Green circles represent electrons. Solid (dashed) curves with double arrowheads indicate the electron hopping that makes AFM (FM) contributions to the NNN super-superexchange. Inserts in (a) and (b) are the local geometrical structure of the Os-O-O-Os paths.

1. **Calculated magnetic exchange parameters of SrCaFeOsO_6_ (SCFOO) and the relaxed structure of Ca_2_FeOsO_6_ (CFOO)**

**
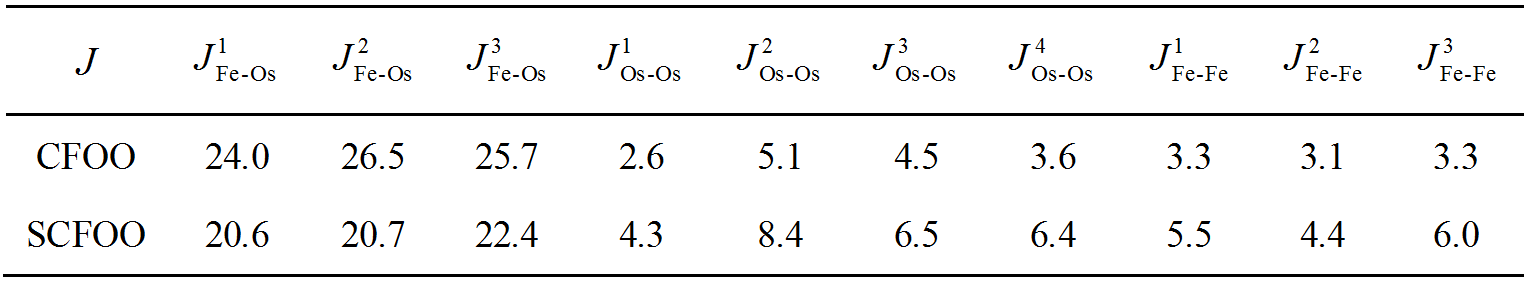
**

Table *I*. Shown are the calculated magnetic exchange parameters of SCFOO and the relaxed structure of CFOO. All J’s are in units of meV.

1. **The magnetic exchange parameters $J_{Fe-Os}^{1}$ in the pseudo-cubic phase of Ca_2_FeOsO_6_ versus the Coulomb interaction U**

| $U_{Fe}^{eff}$ (eV) | $U_{Os}^{eff}$ (eV) | $J_{Fe-Os}^{1}$ (meV) |
| --- | --- | --- |
| 2.0 | 1.0 | 33.4297 |
| 2.0 | 1.5 | 31.6681 |
| 2.0 | 2.0 | 27.2400 |
| 2.0 | 2.5 | 22.5055 |
| 3.0 | 1.0 | 30.3025 |
| 3.0 | 1.5 | 28.0342 |
| 3.0 | 2.0 | 23.7658 |
| 3.0 | 2.5 | 19.7040 |
| 4.0 | 1.0 | 27.0326 |
| 4.0 | 1.5 | 24.4959 |
| 4.0 | 2.0 | 20.6585 |
| 4.0 | 2.5 | 17.3248 |
| 5.0 | 1.0 | 23.7782 |
| 5.0 | 1.5 | 21.2239 |
| 5.0 | 2.0 | 17.9812 |
| 5.0 | 2.5 | 15.2640 |

Table *II*. Shown is the calculated magnetic interaction **$J_{Fe-Os}^{1}$ with**in the pseudo-cubic structure of Ca_2_FeOsO_6,_ versus the Coulomb interaction U. All the calculated *J*’s are positive, meaning that the magnetic interactions of the Fe-O-Os paths are antiferromagnetic regardless of what value of U is assumed.
